# A 10-Year Prospective Study of Socio-Professional and Psychological Outcomes in Students From High-Risk Schools Experiencing Academic Difficulty

**DOI:** 10.3389/fpsyg.2020.01742

**Published:** 2020-07-14

**Authors:** Reda Salamon

**Affiliations:** Bordeaux Population Health Research Center, INSERM U1219, University of Bordeaux, Bordeaux, France

**Keywords:** adolescents, academic performance, outcome, higher education, employment, psychological adjustment

## Abstract

Early academic difficulty reduces the probability of pursuing higher education and has consequences for a wide range of personal and socio-professional outcomes. However, the role of academic performance is often difficult to assess independently from school-based influences. This prospective investigation uses a nested high-risk paradigm to examine the role of personal, familial and school-based factors in the prediction of 10-year outcomes. A sample of 131 secondary school students were selected based on scores in the highest or lowest quartiles in national exams, and both groups were selected equally from regular or low-performing schools. Ten years later, 100 of these individuals participated in a follow-up assessment of academic, socio-professional and personal outcomes. Academic difficulty and specific parental professions were strongly associated with a lower probability of pursuing higher education and with a greater likelihood of part-time or minimum-wage employment. No effect was observed for school status and it did not interact with academic performance in predicting the majority of outcomes. Strategies designed to improve individual academic performance and that address familial difficulties should remain priorities for improving long-term outcomes. The lack of influence of school-based characteristics may indicate the efficacy of strategies aimed at reducing inequalities in resources at the institutional level.

## Introduction

Academic difficulties occurring early in an individual’s education can sometimes evolve into a self-perpetuating series of problems that have long-term consequences at both personal and professional levels. Recent prospective investigations have found that math and reading skills assessed as early as kindergarten predicted math and reading performance in the fifth grade (Rabiner et al., 2016), and that reading difficulty among children 7–8 years old increased by four-fold the risk of secondary school non-completion (Smart et al., 2017). Other longitudinal studies have found that prior academic performance is the single strongest predictor of high school grades (McCarty et al., 2008; Casillas et al., 2012) and a recent meta-analysis of 46 effect sizes from studies including a total of 34,724 students demonstrated that academic success or difficulty in high school is strongly predictive of performance at the university level (Schneider and Preckel, 2017). These consistent findings suggest that difficulties at any phase of education have important consequences for performance at the subsequent phase, from kindergarten through higher education. Added to the persistence of these difficulties over time are the negative consequences outside of academia, as several decades of research have documented the diverse consequences of poor academic performance on socio-professional integration as well as on physical and psychological health (Becker, 1962; Mirowsky and Ross, 2003; Hussenet and Santana, 2004; Lucio et al., 2012; Sörberg Wallin et al., 2018, 2019). While these associations are complex and implicate a large diversity of risk factors, there is clear international consensus that combatting academic difficulty is both a social and economic priority (American Public Health Association, 2010; Organisation for Economic Co-Operation and Development [OECD], 2010).

The strategies applied to reduce academic difficulty early in the educational trajectory have traditionally involved either individual-specific approaches such as tutoring, special classes and other educational interventions, or broader approaches that address inequalities at the institutional level. Although student-specific strategies have demonstrated positive, albeit variable, effects on academic performance and retention (Cohen et al., 1982; Bowman-Perrott et al., 2013; Steenbergen-Hu and Cooper, 2014; Warmbold-Brann et al., 2017), few of these interventions address the frequent sources of social disadvantage that have been shown to predict school performance across all stages of education (Rabiner et al., 2016). These factors notably include family based characteristics such as parental occupation and income, as well as single-parent or migrant family status which not only affect household income but also the resources necessary to provide home-based educational support (Sirin, 2005; Chiu, 2007; Goodman et al., 2010; Najimi et al., 2013; Chittleborough et al., 2014; Camara-Costa et al., 2016; Nonoyama-Tarumi, 2017; Hagan et al., 2018). An important additional consideration is the fact that these different sources of social disadvantage are not randomly distributed in the general population, but rather they are often concentrated within schools, school districts or specific geographic communities (Massey et al., 1991; Jargowsky, 2006; De Langea et al., 2014; Meade, 2014). For this reason, a principal objective of institutional-level strategies is to reduce systemic inequalities and thereby combat academic difficulties for all disadvantaged students (Chittleborough et al., 2014; Jury et al., 2017; Bayless et al., 2018).

Similar to interventions with individual students, school-based approaches appear to have a positive but variable impact on improving academic performance (Farrell et al., 2010; Wilson and Tanner-Smith, 2013; Hahn et al., 2015; Stipek and Chiatovich, 2017; Ruiz et al., 2018). However, long-term outcomes of students with low academic performance have often been difficult to characterize independently from the consequences or benefits of school-based characteristics. For this reason, the present investigation identified secondary school students manifesting academic difficulty or achievement based on their performance on national exams for the core subjects of language and mathematics, and then selected both groups equally from schools with or without a record of poor academic performance (Salamon et al., 2011). Recruited students were then contacted a decade later relative to their pursuit of higher education or employment, as well as concerning their personal living conditions and psychological status. These 10-year outcomes are examined in this investigation as a function of the main effects of baseline academic performance and school type, as well as by family and psychological characteristics. In addition, the interaction of academic performance with school type is examined in order to identify those individuals at potentially highest risk for negative outcomes due to the accumulation of risk factors at both the individual and institutional levels.

## Materials and Methods

### Participants

Between 2005 and 2007, 131 junior high school students experiencing academic difficulty (*n* = 65) or success (*n* = 66) participated in the baseline phase of this study. The age of this original sample was 11.44 years (SD = 0.62) and 54% were females. The two academic performance groups did not differ by sex, *X*^2^(1) = 0.61, *p* > 0.05, but those in academic difficulty were older, *t*(129) = 3.91, *p* < 0.01, and more likely to be behind in their schooling relative to their age *X*^2^(1) = 37.82, *p* < 0.01. Starting in 2017, 104 participants from the original sample were recontacted for the present study, and 100 accepted to participate (average follow-up duration 11.26 years, SD = 0.84, range 10–13 years; average participant age = 23.25 years, SD = 0.93, range 21–26 years).

### Procedure

The procedures for baseline recruitment are described elsewhere (Salamon et al., 2011). In summary, 10 junior high schools in the Bordeaux region of France were selected according to their classification by the French Department of Education as either a regular school (*n* = 5) or as an education priority school (*n* = 5). The official texts defining “Zone d’Education Prioritaire” (Education Priority Zone) status were published in 1981, and specify that its aim is to combat social inequality through reinforced educative actions in schools where both academic and social difficulties are concentrated (Saramon, 2002). Schools obtaining this status must have academic results for their students that are below the national average as well as have students from families experiencing greater socio-economic difficulty than the national average (Bongrand and Rochex, 2016; Stéfanou, 2018). These criteria changed in 2015 (Stéfanou, 2017), after the present sample left the secondary education system. Education priority schools receive additional financial resources and teaching personnel, as well as increased decisional autonomy to obtain academic objectives (Stéfanou, 2018). This study was conducted in full compliance with the World Medical Association’s Declaration of Helsinki. Written informed consent was acquired from both the students as well as their parents before study enrollment, and the study was approved by the regional school district as well as by the French national board for the rights of research participants and data protection (Commission Nationale de l’Informatique et des Libertés).

Within each type of school, two groups of eligible participants were selected based on academic performance. An “academic difficulty” group was constituted based on scores in the lowest 25% on national examinations in both French and mathematics, and an “academic success” group based on scores in the highest 25% in both of these subjects. From these eligible pools of students within each school, individuals were then selected at random until relatively equal numbers of students were selected in each group at each school. These selected participants completed a baseline battery of paper-based sociodemographic and clinical measures. Follow-up measures were administered approximately 10 years later through a telephone interview and a web-based sociodemographic and clinical battery.

### Sociodemographic Measures (Baseline Assessment Only)

Diverse individual characteristics were assessed at baseline, including age, sex, advanced or late in academic year relative to age, dual versus single-parent household and, for mothers and/or fathers, profession, place of birth (France or other country), and native language (French or other language).

### Clinical Measures (Baseline and Follow-Up Assessments)

Baseline depression levels were assessed using the French version of the 27-item Children’s Depression Inventory (CDI); (Kovacs and Beck, 1977; Moor and Mack, 1982). The CDI evaluates depressive symptoms (sad mood, anhedonia, and items specific to academic and social domains) on a three-point scale, and has internal consistencies of 0.86 for the original instrument and 0.82 in the present sample. At follow-up, depression levels were assessed by the 21-item Beck Depression Inventory (BDI); (Beck et al., 1961; Bourque and Beaudette, 1982). The BDI evaluates diverse emotional, behavioral and cognitive symptoms of depression in adults on four-point scales, and has internal consistencies of 0.86 for the original instrument and 0.88 in the present sample. Baseline anxiety was measured by the State-Trait Anxiety Inventory (STAI) for Children, a 20-item measure developed to assess anxiety in elementary school children using four-point scales (STAI; Spielberger, 1973; Vila et al., 1999), and its adult version (Schweitzer and Paulhan, 1990) was used at follow-up. The STAI has strong psychometric qualities and internal consistencies for trait anxiety (state anxiety was not assessed in this study) of 0.90 for either the original child or adult versions (0.84 and 0.92, respectively, in the current sample). Self-esteem was assessed at both baseline and follow-up by the 10-item French version of the Rosenberg Self-Esteem Scale (Rosenberg, 1965; Vallières and Vallerand, 1990; GrayLittle et al., 1997; Yarcheski et al., 2003). These items evaluate general self-esteem on four-point scales, and internal consistencies reported for the original instrument were 0.87 in adolescents and 0.88 in young adults. For the present sample, internal consistencies for this instrument were 0.76 at baseline (adolescents) and 0.87 at follow-up (adults).

### Socio-Professional Outcomes (Follow-Up Assessment Only)

Professional and education outcomes included mutually exclusive categories of education status (current enrollment, year of study, and highest diploma obtained), occupational status (unemployed, apprentice or intern, and full or part-time employed), and living conditions (living alone, with parents or relatives, with partner or spouse, or with roommates).

### Statistical Analyses

Linear and logistic regression analyses were used in the prediction of continuous or dichotomous outcomes, respectively. The principal predictor variable in these analyses was either baseline academic status (difficulty or success), or type of school (normal or education priority), adjusted for age and sex. All outcomes variables were those assessed at the 10-year follow-up only. A power analyses demonstrated that the final sample size of 100 individuals provided above 0.80 power for detecting small to medium effects (*f*^2^ = 0.12) with three predictors (age, sex, and either academic status or type of school) at *p* < 0.05. All analyses were conducted using SPSS version 23.

## Results

From the original baseline sample of 131 junior high school students, 104 were located and contacted 10 years later. One hundred of these individuals agreed to participate in the second wave of the survey (Table 1). Contact information could not be found for 27 persons, or 87% of the 31 non-participating individuals. A significantly greater proportion (81%) of non-participants were in the academic difficulty group at baseline *X*^2^(1) = 15.64, *p* < 0.001. These individuals were significantly less likely to have either a mother or father in higher-level professions, *X*^2^(1) = 6.75, *p* < 0.05, and more likely to have foreign-born parents *X*^2^(1) = 4.54, *p* < 0.05. At baseline, these individuals also reported significantly higher depressive symptomatology, *t*(129) = 2.33, *p* < 0.05.

**TABLE 1:** Baseline characteristics of participants recontacted and non-recontacted at 10 years.

Baseline variable	Recontacted (*n* = 100)			Non-recontacted (*n* = 31)			*p*
Academic difficulty	40%			81%			0.001
Education priority school	42%			58%			ns
Behind for age	19%			32%			ns
In advance for age	7%			0%			ns
**Familial characteristics**							
Single parent household	23%			32%			ns
Higher profession (father)	39%			19%			ns
Higher profession (mother)	39%			16%			0.028
Parental birth place	89%			73%			0.042
Parental maternal language	89%			77%			ns

	**M**	**SD**	**%**	**M**	**SD**	**%**	***p***

**Sociodemographic characteristics**							
Age	11.42	0.61		11.52	0.68		ns
Sex (% female)			56%			48%	ns
**Psychological characteristics**							
Anxiety	31.89	6.70		32.65	6.46		ns
Depression	8.21	5.61		11.01	6.58		0.021
Self-esteem	31.98	4.31		30.41	5.00		ns

*Academic difficulty = students with performance levels in the lowest 25% on national exams for both French and mathematics (compared to students in the highest 25% for both subjects); Education priority school = schools characterized by average student academic performance that is significantly lower than the national average (compared to regular schools).*

**FIGURE 1:**
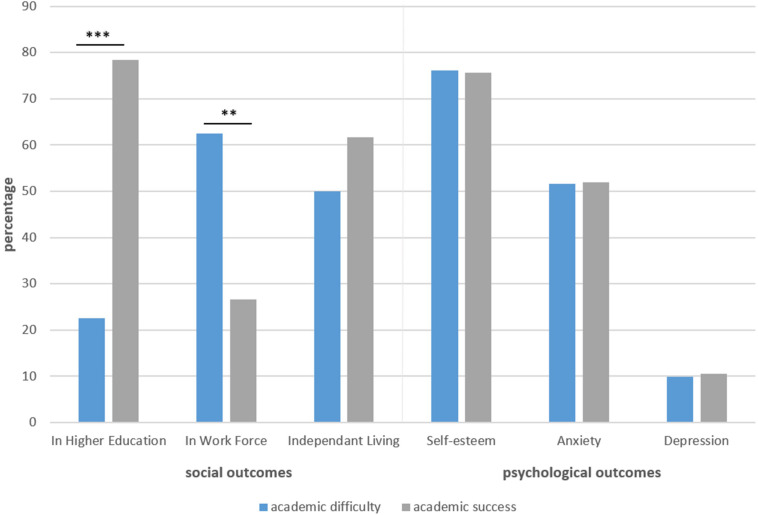
Academic difficulty or success in adolescence as a predictor of selected 10-year socio-professional and psychological outcomes. ^∗∗^*p* < 0.01 and ^∗∗∗^*p* < 0.001.

For the participating sample (*n* = 100), follow-up outcomes were first examined by baseline academic status (Table 2). Individuals in academic difficulty (versus achievement) were more likely to have a socio-professional status other than pursuing higher education (OR = 12.82, 95% CI = 4.58–35.87; Nagelkerke *R*^2^ = 0.37, χ^2^ = 32.66, *p* < 0.001; 78.0% correct classification). They were also more likely to already be in the workforce (OR = 4.28, 95% CI = 1.74–10.53; Nagelkerke *R*^2^ = 0.17, χ^2^ = 13.20, *p* < 0.01; 69.0% correct classification). Of the 31 employed individuals who were not pursuing higher education, the nature of employment most frequently involved unskilled and semi-skilled work or service-oriented positions (84%). Fourteen of these individuals (44% of the working sample) were employed on a part-time basis only, with an average gross income of 1066 euros. Individuals employed full-time (*n* = 17) earned 1550 euros on average, with 44% earning minimum wage. Among all participants who did pursue higher education, those who experienced academic difficulty at baseline were less advanced in their attainment of degrees, *X*^2^(1) = 8.96, *p* < 0.05. However, no significant association of baseline academic difficulty was observed relative to anxiety or depression symptomatology, or self-esteem levels. Figure 1 provides a visual summary of these findings for a selection of primary outcomes.

**TABLE 2:** Baseline academic difficulty (versus academic success) as a predictor of 10-year outcomes (*n* = 100).

10-year outcome	*B*	SE	OR	*T*	*p*
**Education/professional status**					
Current university studies	−2.551	0.525	12.819		0.000
Apprentice or trainee	−0.115	0.945	0.891		ns
Paid employment	1.454	0.459	4.281		0.002
Non-employment	−1.520	0.866	0.219		ns
**Housing status**					
Living alone	−0.103	0.594	0.902		ns
Living with family	0.639	0.443	1.895		ns
Living with partner	0.640	0.552	1.896		ns
Living with friends/roommates	1.734	0.685	0.177		0.011
**Psychological characteristics**					
Depression	0.450	1.433		0.314	ns
Anxiety	1.166	2.267		0.514	ns
Self-esteem	−0.548	0.941		−0.582	ns

*Academic difficulty = students with performance levels in the lowest 25% on national exams for both French and mathematics (compared to students in the highest 25% for both subjects).*

Ten-year outcomes were then examined as a function of the type of schools in which students were enrolled (regular schools, or education priority schools benefiting from additional resources due to lower academic success rates). As demonstrated by Table 3, school status had no main effect relative to any of the follow-up outcomes in the socio-professional, housing, or psychological domains. When the interaction of levels of academic status with types of school was examined, again no association was found for follow-up outcomes with the exception that living with a spouse or partner was concentrated in majority among individuals who were in both academic difficulty and education priority schools at baseline (OR = 24.49, 95% CI = 1.81–332.05; Nagelkerke *R*^2^ = 0.24, χ^2^ = 15.78, *p* < 0.01, 81.0% correct classification).

**TABLE 3:** Baseline education priority school (versus standard school) as a predictor of 10-year outcomes (*n* = 100).

10-year outcome	*B*	SE	OR	*T*	*p*
**Education/professional status**					
Current university studies	0.721	0.427	2.057		ns
Apprentice or trainee	−0.503	0.911	0.605		ns
Paid employment	0.405	0.423	1.500		ns
Non-employment	0.196	0.765	1.216		ns
**Housing status**					
Living alone	0.295	0.413	1.344		ns
Living with family	−0.933	0.622	0.393		ns
Living with partner	1.061	0.556	2.888		ns
Living with friends/roommates	−0.628	0.518	0.534		ns
**Psychological characteristics**					
Depression	0.534	1.357		0.394	ns
Anxiety	2.732	2.132		1.281	ns
Self-esteem	−0.403	0.891		−0.452	ns

*Education priority school = schools characterized by average student academic performance that is significantly lower than the national average (compared to regular schools).*

In addition to the main effects of personal or school academic status, other baseline variables were examined for their association with 10-year outcomes. The sex of students was unrelated to socio-professional or housing status. However, females reported lower self-esteem at follow-up, *B* = −2.75 *p* < 0.01, as well as higher levels of both anxiety, *B* = 5.67, *p* < 0.01, and depressive symptomatology, *B* = 3.40, *p* < 0.05, than did males. Students who were behind in academic progress relative to their age at baseline were more likely to have a socio-professional status other than pursuing higher education at follow-up (OR = 7.97, 95% CI = 1.95–32.52; Nagelkerke *R*^2^ = 0.17, χ^2^ = 13.55, *p* < 0.01, 67.0% correct classification), while those having a father in a higher-level profession were more likely to pursue higher education (OR = 5.96, 95% CI = 2.25–15.79. Nagelkerke *R*^2^ = 0.24, χ^2^ = 19.32, *p* < 0.001, 67.7% correct classification) and less likely to be in the workforce (OR = 0.21, 95% CI = 0.83–0.55. Nagelkerke *R*^2^ = 0.18, χ^2^ = 14.07, *p* < 0.01, 65.7% correct classification). Having a mother with a higher-level profession was also associated with increased probability of pursuing higher education (OR = 4.83, CI = 1.91–12.22. Nagelkerke *R*^2^ = 0.20, χ^2^ = 16.20, *p* < 0.001, 67.0% correct classification), as well as with higher self-esteem at follow-up, *B* = 1.892, *p* < 0.05. Living in a single-parental family was associated with lower self-esteem, *B* = −2.114, *p* < 0.05, but with no other follow-up outcome.

## Discussion

Academic difficulty early in the individual’s education has important consequences for later academic success as well as for a range of personal outcomes (Pustjens et al., 2004; McCarty et al., 2008; Casillas et al., 2012; Rabiner et al., 2016; Schneider and Preckel, 2017; Smart et al., 2017). While strategies used to combat academic difficulty are often delivered to individual students, other approaches attempt to address inequalities between schools that vary in overall levels of student performance and socioeconomic resources. A higher concentration of social disadvantage relative to parental employment, income or family structure have direct associations with academic difficulty in youth (Rabiner et al., 2016). This study therefore attempted to clarify the role of early academic difficulty separately from school-based characteristics using a nested, behavioral high-risk paradigm. Students in secondary school experiencing either difficulty or mastery of two core academic subjects were selected in approximately equal proportions from schools designated as having either regular or below-average levels of academic achievement. The 10-year outcomes of these individuals were then examined as a function of academic performance and school status, as well as for the interaction of these variables, and relative to several personal or familial characteristics.

A first notable finding concerns the nature of the participating sample at follow-up. Of the initial baseline sample of 131 students, 100 participated in the survey 10 years later. Despite this high acceptance rate, the majority of students (*n* = 27) who did not participate no longer lived at the same address as when in secondary school and they were not contactable through an exhaustive search of electronic databases and social media. These individuals were also considerably more likely to have been in academic difficulty at the baseline inclusion, as well as to have foreign-born parents who did not have higher-level professions. While the association of migrant status with academic difficulty is inconsistent (Fuligni, 1997; Hagan et al., 2018), families with lower income are at greater risk for such problems (Sirin, 2005; Chiu, 2007; Goodman et al., 2010; Najimi et al., 2013; Chittleborough et al., 2014; Dietrichson et al., 2017; Hasl et al., 2019). The broader role that socio-economic status plays may be both direct (through determining access to educational resources or materials, and providing means for academic support), or indirect through the lack of integration of parents into the school system where they would normally gain knowledge of its potential resources (Sirin, 2005; Hill and Tyson, 2009; Vellymalay, 2012; Benner et al., 2016; Dietrichson et al., 2017). Targeted interventions through schools that integrate and train parents as educational partners can improve the academic performance of children from even the most disadvantaged socioeconomic backgrounds (Dietrichson et al., 2017). In the present study, students who were not contactable and therefore who did not participate in the follow-up may have been at greatest risk for continued academic difficulty due to the lack of such resources.

Concerning students who participated in the 10-year follow-up, a primary but expected finding was that individuals in academic difficulty as adolescents were far less likely to pursue higher education. Moreover, when they did pursue their education, they were more often behind in their attainment of a diploma or degree relative to other students. Academic difficulty also predicted a greater likelihood of entering the workforce instead of pursuing university education. The nature of this employment most often included unskilled or semi-skilled jobs, and a large minority (44%) were employed only part-time and without pursuit of their education in parallel. As for income, 44% of full-time workers earned the minimum wage and the average income of part-time workers was only 1066 euros. Considering that the French poverty threshold in 2018 was 1026 euros for a single adult (Argouarc’h and Picard, 2018), it is likely that the professional situation of a large majority of these individuals was insufficient to sustain independent living needs. The association of early academic difficulty or school drop-out with the probability of precarious or lower-paying employment is well-documented (Rumberger, 1987; Stillwell, 2009; UK Office for National Statistics, 2011; Rabiner et al., 2016; Smart et al., 2017), leading to fewer socio-economic resources for the individual and potentially to reduced educational opportunities for their own children (Sirin, 2005; Dietrichson et al., 2017). The present findings therefore underscore the need for intensive efforts early in the educational trajectory to address the inter-generational persistence of academic difficulty and social disadvantage.

In contrast to findings for individual academic performance, school status did not appear to influence the pursuit of higher education or other 10-year outcomes. Importantly, only very limited evidence was found for the interaction of school type with academic achievement group. In understanding these findings, it is important to interpret them within the context of the French public school system and relative to national educational policies. In particular, French schools classified as “education priority institutions” receive additional resources from the government, including increased financial support, additional staff to lower teacher-to-student ratios, and increased autonomy in developing strategies for improving academic performance (Stéfanou, 2018). The lack of findings for school type in this context may therefore indicate the potential success of such governmental policies put into place to address school inequalities and combat academic difficulty at the institutional level. The lack of interaction between school status and individual academic performance indicates that students who are in difficulty or who are succeeding academically in lower-performing schools have no less of a chance of pursuing higher education than if they attended a regular-performing school. While the success of this French policy cannot be fully verified by the present investigation alone, confirmation of its efficacy would provide an important model for overcoming certain sources of educational inequality.

The strengths of this study include its prospective design as well as its application of a high-risk paradigm that nested high or low academic performance groups within high or low risk schools. However, the present findings should be interpreted in light of both conceptual characteristics and methodological limitations. A first consideration is that the non-participation at follow-up of nearly one-fourth of the original sample, individuals who were more often in academic difficulty at baseline, may in fact underestimate the frequency of negative outcomes among students in the high-risk group. The moderate sample size at follow-up may also have hindered the identification of more subtle but important associations between the risk groups and specific socio-professional or personal outcomes. In additional to the strategies used in France to address inequalities between schools, the findings should be interpreted relative to its higher education system that has important differences with other developed countries. A long-standing tradition in France is that all students graduating high school are allowed to enroll in French public universities regardless of prior grades or test scores. Other educational systems may therefore observe even greater disparities between individuals who experience early academic difficulty or success if university enrollment is based on competitive selection criteria. While France has recently adopted reforms for universities that place restraints on the choices of students for certain academic disciplines, these changes were adopted after the present sample participated in the 10-year follow-up. The coming years will clarify if these new reforms exacerbate the negative consequences of early academic difficulty relative to the pursuit of higher education. Nonetheless, the present findings underscore both the importance of early intervention relative to academic difficulties as well as the need for further prospective research capable of investigating the independent influences of frequently confounded risk factors.

## Data Availability Statement

The raw data supporting the conclusions of this article will be made available by the authors, without undue reservation.

## Ethics Statement

The study was reviewed and approved by CCPPRB Ethics Review Board, and the Académie de Bordeaux. The patients/participants provided their written informed consent to participate in this study.

## Author Contributions

RS was solely responsible for the design and conceptualization of the study, for all data collection and analyses, and for manuscript preparation.

## Conflict of Interest

The author declares that the research was conducted in the absence of any commercial or financial relationships that could be construed as a potential conflict of interest.
